# Enzymatic Reaction-Related Protein Degradation and Proteinaceous Amino Acid Metabolism during the Black Tea (*Camellia sinensis*) Manufacturing Process

**DOI:** 10.3390/foods9010066

**Published:** 2020-01-08

**Authors:** Yiyong Chen, Lanting Zeng, Yinyin Liao, Jianlong Li, Bo Zhou, Ziyin Yang, Jinchi Tang

**Affiliations:** 1 Tea Research Institute, Guangdong Academy of Agricultural Sciences & Guangdong Provincial Key Laboratory of Tea Plant Resources Innovation and Utilization, Dafeng Road 6, Tianhe District, Guangzhou 510640, China; chenyiyong@gdaas.cn (Y.C.); skylong.41@163.com (J.L.); zhoubo@gdaas.cn (B.Z.); 2 Key Laboratory of South China Agricultural Plant Molecular Analysis and Genetic Improvement & Guangdong Provincial Key Laboratory of Applied Botany, South China Botanical Garden, Chinese Academy of Sciences, Xingke Road 723, Tianhe District, Guangzhou 510650, China; zenglanting@scbg.ac.cn (L.Z.); honey_yyliao@scbg.ac.cn (Y.L.); zyyang@scbg.ac.cn (Z.Y.); 3 Center of Economic Botany, Core Botanical Gardens, Chinese Academy of Sciences, Xingke Road 723, Tianhe District, Guangzhou 510650, China

**Keywords:** amino acids, black tea, *Camellia sinensis*, fermentation, volatile compounds

## Abstract

Amino acids contribute to the nutritional value and quality of black tea. Fermentation is the most important stage of the black tea manufacturing process. In this study, we investigated protein degradation and proteinaceous amino acid metabolism associated with enzymatic reactions during fermentation in the black tea manufacturing process. The results showed that the concentrations of both protein and free amino acids decreased during fermentation. We also confirmed that proteins were broken down into free amino acids by artificially synthesized dipeptide benzyloxycarbonyl glutamyl-tyrosine (Z-Glu-Tyr). Metabolites of the amino acid metabolic pathway increased significantly during fermentation. Furthermore, we confirmed that free amino acids were degraded to volatile compounds in a tracer experiment with the isotope precursor. These results provide information that will help black tea manufacturers improve the quality of black tea.

## 1. Introduction

Amino acids (AAs) have many important physiological and nutritional functions in humans, which are not only closely related to body growth and development, but also health and disease. Traditionally, 20 proteinaceous amino acids involved in constructing protein skeletons are classified as nutritionally essential or nonessential amino acids [1]. In plants, amino acids perform various critical functions. In addition to their roles in protein synthesis, various physiological processes in plants have been reported to be related to amino acids [2,3,4,5,6,7]. First, amino acids are important nutrients and regulatory substances for plant growth and development, and act as intracellular pH regulators. Second, metabolic energy or redox power generation in plants is related to amino acids metabolism. Finally, amino acids are plant resistant factors against both abiotic and biotic stress. For example, amino acids have been reported to have significant effects on pathogen infection. On one hand, amino acid metabolites can serve as important defense compounds, on the other hand, they also act as indispensable nitrogen sources for many biotrophic pathogens [8,9,10]. Furthermore, the role of amino acids during signaling in plants has been discussed [4,5,6,7,8,9,10,11]. Essential amino acids significantly contribute to the nutritional quality of plant-originated foods. Fruit taste might be influenced by some free amino acids. A well-known example is the contribution of glutamate (L-Glu), which has delicious savory taste, to the umami flavor of fruits and vegetables [12]. Furthermore, alanine (Ala) and lysine (Lys) contribute to the sweetness of food, while phenylalanine (L-Phe) and tyrosine (Tyr) taste bitter [13].

A close relationship exists between amino acid metabolism and carbohydrate metabolism. The substrates used for amino acid synthesis are derived from carbohydrate metabolites, and some products of amino acid degradation can act as energy substances in the citric acid cycle. Furthermore, amino acid metabolism is closely related to nitrogen absorption and utilization. Inorganic nitrogen absorbed from plant roots can be synthesized into organic molecules through the glutamate synthesis pathway, and all other nitrogen-containing organic compounds are synthesized from glutamate and glutamine [14]. Additionally, amino acid metabolism products are important substances for protein synthesis and secondary metabolism.

Tea manufactured from *Camellia sinensis* leaves as the raw material is the most consumed plant-derived beverage globally. Leaves, flowers, fruits, roots, stems, and even some whole wild plants can be processed into teas, known as tea substitutes in China [15]. Though tea-substitute plant resources and related products are common in China, kudingcha and *Lycium barbarum* tea are the most common tea substitutes made from leaves [16,17]. However, only *Camellia sinensis* tea has a long history of consumption worldwide. The production of *Camellia sinensis* has important value in agriculture and commerce, owing to its peculiar flavor and nutritional functions. Amino acids, caffeine, and other substances in tea leaves endow tea with many physiological and pharmacological properties [18]. The relationship between amino acid content and tea quality has been studied by Yang et al. (2012) [19]. Free amino acids have been reported to make important contributions to the quality and function of tea leaves. Some free amino acids are key precursors of tea aroma compounds, for example, the aromatic amino acids can be converted into the aroma components of tea [19,20,21]. Theanine is the most abundant and most characteristic free amino acid in tea leaves. Theanine not only affects the taste and aroma of tea, but also has many physiological functions in the body. The functions of theanine in relaxation and against cancer have been reported [20,22].

Manufacturing practices play a decisive role in the formation of optimum tea flavor and quality. Tea leaves used for manufacturing are usually freshly picked, and can comprise the bud or the first two leaves of the tea shoot. After harvesting, the tea leaves are immediately transported to a manufacturing factory for subsequent processing. According to the different degrees of fermentation in tea processing, manufactured tea leaves are generally classified into three types; tea leaves without fermentation are processed into green tea, those that are partially fermented are processed into oolong tea, and those that are fully fermented are processed into black tea. Enzymatic oxidation is the key biochemical reaction in all types of tea processing, and is positively correlated with the degree of fermentation. In black tea processing, the enzymatic oxidation process during fermentation determines the quality of black tea [23]. Free amino acids contribute significantly to the flavor features of black tea, and their contents change significantly during fermentation. Previous research has shown that the concentrations of some free amino acids—such as glutamic acid, glutamine, leucine, serine, isoleucine, phenylalanine, threonine, and theanine—decrease appreciably during fermentation, while other amino acids show little change [24]. In 1965, Wickremasinghe and Swain reported that free amino acid contents were overall decreased during black tea fermentation [25]. These results suggested that free amino acids may be converted into other metabolic components during fermentation. Several studies have suggested that some important volatile aroma components in black tea are derived from the conversion of amino acids [26,27]. However, little is known about the metabolism of free amino acids during black tea fermentation. In the present study, we aimed to analyze the changes in free amino acid profiles and contents at different enzyme reaction stages of the black tea manufacturing process. We also investigated whether protein degradation contributed to the changes in free amino acids and verified the conversion of free amino acids into the volatile aroma components of black tea during fermentation.

## 2. Materials and Methods

### 2.1. Plant Materials and Black Tea Manufacturing

**Figure 1:**
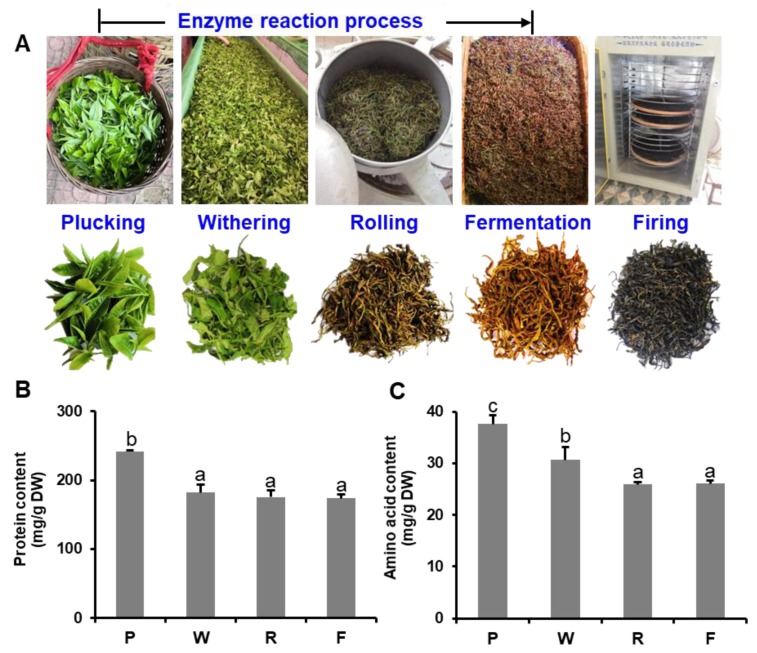
Changes of protein content and amino acids content during the enzymatic reaction of black tea manufacturing process. P, Plucking; W, Withering; R, Rolling; F, Fermentation. (**A**) Five process stage of black tea (*Camellia sinensis*) manufacturing; (**B**,**C**) the changes of protein content and total amino acids content during the enzymatic reaction of black tea manufacturing process. Bars indicate the means ± S.D. (*n* = 3) of three biological replicates, and bars with different letters are significantly different at *p* ≤ 0.05 according to Duncan’s multiple range test.

Tea leaf samples used in this study comprised one bud and two leaves from *C. sinensis* cv. Yinghong No.9. Tea plants were planted in Yingde Tea Experimental Station of Tea Research Institute, Guangdong Academy of Agricultural Sciences (23° N, 113° E, Yingde, China). Samples were collected in July 2018, and the tea leaves were immediately transported to the manufacture factory, the method for black tea manufacture was according to the general processing (Figure 1A). Fresh tea leaves (plucking) were withered indoor at temperature about 25 °C for 10 h [28]. The withered tea leaves (withering) were then rolled 40 min using a roller (6CR-10, Fuyang Machinery Co. Ltd., Hangzhou, China) at room temperature. After rolling (rolling), the tea leaves were placed in an environment control room at a temperature of 25 °C, and relative humidity of 90% for fermentation. After 4 h, the fermented tea leaves (fermentation) were transferred into a tea-firing roller machine (JY-6CHZ-7B, Fujian Jiayou Tea Machine Intelligent Technology Co. Ltd., Anxi, China), parched at 250 °C for 30 min to fix sample (firing). Finally, the tea leaves were dried using a hot air drier at 120 °C (JY-6CHZ-7B, Fujian Jiayou Tea Machine Intelligent Technology Co. Ltd., Anxi, China). Tea samples were collected at each stage and immediately frozen with liquid nitrogen. Three independent biological replicates were processed for each step of black tea manufacturing.

### 2.2. Determination of Total Amino Acids and Total Soluble Protein Content at Each Step of Black Tea Manufacture 

Determination of total free amino acids content in black tea leaves was used the method of ninhydrin colorimetry [29]. Tea leaf samples (0.5 g) were finely crushed with a mortar and pestle, and extracted with 10 mL distilled water in boiling water bath for 1 h. After cooling to room temperature, the mixture was centrifuged using an Allegra 64R centrifuge (Beckman Coulter Inc., Fullerton, CA, USA), at 10,000× *g* for 10 min. Ninhydrin reagent (2%, *w*/*v*) and phosphate buffer (pH 8.0), respectively 0.5 mL, were mixed with 1 mL extracted supernatant, and reacted in a boiling-water bath for 15 min. the reaction mixture was transferred to a 10 mL volumetric flask and made up to volume with distilled water, before standing at room temperature for 10 min. The absorbance of each sample was measured using an ultraviolet spectrophotometer (MetashUV-5200, Shanghai Metash Instruments Co., Ltd., Shanghai, China) at 570 nm. Glutamate was used as the standard substance to construct the standard curve. The protein content was measured according to the Bradford assay method, and the bovine serum albumin (BSA) calibration line was established to quantify the protein content [30]. The moisture contents of samples at each step of the black tea manufacturing were determined using standard method GB 5009.3-2010 [31]. Total amino acids and protein content were expressed as dry weight (DW).

### 2.3. Free Amino Acid Content Analysis for Each Step of Black Tea Manufacture

To analyze the content of each free amino acid in tea samples, the method previously described by Mei et al. (2016) was used [32]. Tea leaves from each process step during black tea manufacture were finely powdered with a mortar and pestle. One hundred milligrams tea leaves powder was placed in a 2 mL centrifuge tube and 0.5 mL of pre-cooled methanol used as the extraction solution added. the mixture was vortex 2 min using a vortex mixer (Scientific Industries, Inc., Suffolk, MA, USA), and subjected to ultrasonic extracted in an ice bath for 15 min. Chloroform (0.5 mL) and distilled water (0.2 mL) were added to the tube and mixed. Centrifugation followed at 5000× *g*, 4 °C for 10 min, with the extraction solution in the tube undergoing phase separation. The upper layer was obtained, and dried by a vacuum centrifugal enrichment at 45 °C. Next, 5% sulfosalicylic acid solution was added to the tube to redissolve the dried crude extraction, followed by filteration through a 0.45 μm nylon filter membrane. An automatic amino acid analyzer (Sykam S-430D, Eresing, Germany) was used to analyze the concentrations of each free amino acid in the samples. The mobile phase was a physiological Li C4 system containing lithium citrate buffer at pH 2.9, 4.2, and 8.0. According to the operating instructions of the instrument, the flow velocity of the mobile phase was set as 0.45 mL/min, and the flow velocity of the derived reagent ninhydrin was set as 0.25 mL/min. The injection volume was 50 μL. The temperatures of the sodium cation-exchange column and post-column reaction equipment were respectively set at 38 °C and 130 °C. UV-Vis detection was used to measure the peaks of each free amino acids at wavelengths of 570 nm and 440 nm. The auto-sampler temperature was set at 5 °C.

### 2.4. Simulation of Enzyme Reaction Step during Black Tea Manufacturing Processes and Analysis of Free Amino Acid Content 

Fleshly plucked tea leaves were completely crushed with liquid nitrogen to simulate the rolling process of black tea manufacture. Non-crushed tea leaves were used as control. Tea samples were placed in an artificial incubator for 6 h of fermentation, and the temperature was set at 25 °C with a relative humidity of 95%. Tea samples were collected at 0 h and 6 h and immediately frozen with liquid nitrogen. The total amino acid and protein contents were measured using the methods mentioned above.

### 2.5. Investigation of Protein Degradation during Black Tea Fermentation with Artificially Synthesized Dipeptide Benzyloxycarbonyl Glutamyl-Tyrosine (Z-Glu-Tyr) 

Tea leaves were completely crushed with liquid nitrogen, finely powdered tea leaves (100 mg) and 25 μL 50 g/L dipeptide Z-Glu-Tyr were mixed together in a centrifugal tube, then before fermenting in an incubator for 6 h at 25 °C and a relative humidity of 95%. The control comprised crushed tea leaf power and 25 μL 50 g/L dipeptide Z-Glu-Tyr fermented separately for 6 h and then mixed. Each free amino acid was extracted and detected using the methods described above.

### 2.6. Analysis of Black Tea Aroma Compounds during the Manufacturing Processes

Aroma compounds of black tea during the manufacturing processes were analyzed, and the method referred to the research reported by Zeng et al. (2019) [33]. Tea leaf sample powder (0.5 g) and extracting buffer (dichloromethane (2 mL) contain 0.5 nmol ethyl *n*-decanoate as an internal standard) were added into a 5 mL glass vial, and the samples were extracted overnight on an orbital shaker. The extraction solution was then passed through a sodium sulphate anhydrous column to remove residual water and collected in a 2 mL glass tube. The tube was transported to a termovap sample concentrator (NDK200-1, MIU Instruments Co., Ltd., Shanghai, China). All samples were concentrated to 200 μL, transferred to GC-MS sample bottles, and the aroma compounds in these samples were analyzed used the instrument GC-MS QP2010 SE (Shimadzu Corporation, Kyoto, Japan) according to manufacturer instructions. A SUPELCOWAX 10 column (30 m × 0.25 mm × 0.25 μm, Supelco Inc., Bellefonte, PA, USA) was equipped to separate aroma compounds. The GC injection port temperature was set at 230 °C and held for 1 min. Splitless control mode was selected. High purity helium gas was used as the carrier, and the flow velocity was set at 16.667 μL/s. As for the temperature program settings, the initial temperature of GC oven was set at 60 °C, helding for 3 min, and then ramped up to a final temperature of 240 °C at a rate of 0.067 °C/s, helding for 30 min. For MS program, full scan acquisition mode was selected for mass spectra collection, starting at *m*/*z* 40, and ending at *m*/*z* 200. Identification and quantitative analyses of benzaldehyde, benzeneacetaldehyde, benzyl alcohol, and phenylethyl alcohol were conducted by direct comparison with authentic standards and calibration curves.

### 2.7. Investigation of the Amino Acid Catabolism during Black Tea Fermentation with Supplementation of [^2^H_8_] L-phenylalanine

Fresh tea leaves (0.2 g) were crushed with liquid nitrogen in a mortar. Then 100 μL of 10 g/L labeled mixed [^2^H_8_] L-phenylalanine solution was added, followed by fermentation in an incubator at 25 °C for 6 h. The control was tea leaf powder without fermentation. After fermentation, the samples were collected and extracted with dichloromethane. The volatiles among labeled phenylpyruvic acid products were analyzed by GC-MS. The method for volatiles analysis products of tea leaf samples is described above. Three independent experiments were performed.

### 2.8. Statistical Analysis

All experiments in the study were repeated three times, and Excel 2010 and SPSS Statistics 23.0 software were used for calculation and statistical analysis. The experimental results were expressed in the form of mean ± standard deviation (S.D.). Student’s *t*-test was used to calculate significant differences between two treatments (* *p* ≤ 0.05, ** *p* ≤ 0.01). For three or more treatments, the differences were calculated by one-way ANOVA followed by Duncan’s multiple comparison tests. Significant differences between groups were defined at the probability level of 5% (*p* ≤ 0.05).

## 3. Results and Discussion

### 3.1. Changes in Proteins and Free Amino Acids during the Black Tea Manufacturing Process

Generally, teas are divided into different categories according to different processing technologies, and the difference in fermentation degree is the most important characteristic of all types of teas. The three major categories of tea, in order of increasing degrees of fermentation, are green tea, oolong tea, and black tea [20]. In an orthodox manufacturing process, fresh tea leaves are picked from the shoots of tea plants, after four independent processing steps—namely, withering, rolling, fermentation, and drying—black tea is produced (Figure 1A). The withering process during black tea manufacturing results in partial moisture removal (Table S1). In this stage, the degree and duration of withering must be well controlled to ensure that the tea leaves are in a good physiological state, which is suitable for the subsequent manufacturing stages. Many biochemical reactions that seem to affect product quality, such as the breakdown of proteins to amino acids, have been reported to occur during the withering stage [24]. The cellular compartments of tea leaves are disrupted during the subsequent processing stage of rolling, with the cytoplasmic polyphenol oxidase coming into contact with catechin substrates as monomers in the vacuole. This is followed by the fermentation step, during which the most important reaction is the polyphenol oxidase and peroxidase catalyzed oxidation of tea polyphenols. In the biochemical reaction of black tea fermentation, a large amount of heat is released, which might cause unwanted secondary metabolism reactions and, therefore, affect the quality of tea components. Therefore, the temperature during the fermentation process must be controlled. These biochemical reactions during fermentation are terminated during subsequent drying, with some flavor characteristics formed at this stage.

Tea protein has recently been reported to have biological functions, such as antioxidant, antimutation, and radiation protection functions [34]. Previous studies have mainly focused on the metabolic transformation process of tea polyphenols and catechins, with tea proteins having seldom been investigated until recently. In the present study, the soluble protein content of tea leaves decreased during the black tea manufacturing process (Figure 1B). The main reason for this decrease in protein content was tea leaf protein degradation. Black tea manufacture processes from fresh tea leaf plucking to rolling also involved partial fermentation. During these processes, tea leaf withering and crushing induced oxidation reaction. The cellular compartments of tea leaves were disrupted, and proteases in plastids were activated to more easily capture proteins.

In theory, due to protein degradation, the free amino acid content should increase during the black tea manufacturing process. However, the total amino acid content decreased during the manufacturing process (Figure 1C). Thirty free amino acids were detected in tea leaves during the black tea manufacturing process. Notably, 15 free amino acids (including threonine, serine, valine, cysteine, methionine, isoleucine, leucine, tyrosine, and phenylalanine) showed significantly increased contents during withering (*p* < 0.05), but decreased contents in subsequent steps of the manufacturing process (Table 1). The free amino acids formed were likely important for determining tea quality [27]. Total free amino acid content usually accounts for 1–4% of the dry weight of tea leaves, and the types of free amino acids and their proportion in tea are closely related to tea aroma and taste [35]. Free amino acids not only contribute to a different oral taste of tea, such as bitterness, sweetness, and umami-like of tea, but can also be converted into other quality components [36]. The plucked shoot tips of the tea plant can be considered senescent plant tissues. The enzymatic breakdown of proteins is a well-known feature of senescence [37], and the increase in free amino acids in tea leaves during withering demonstrates this general phenomenon. The overall decrease in free amino acid content that occurs during fermentation suggests that they are converted into other substances. In previous research, results have suggested that free amino acids are partially converted into volatile compounds in tea. Some of these volatile compounds are considered to be important constituents of tea aroma. During the drying stage, amino acids can lead to the formation of aromatic substances under heating conditions, such as indoles, alcohols, and aldehydes after a series of complex reactions [38]. 

### 3.2. Protein Degradation during Black Tea Fermentation with Artificially Synthesized Dipeptide Benzyloxycarbonyl Glutamyl-Tyrosin (Z-Glu-Tyr) 

**Figure 2:**
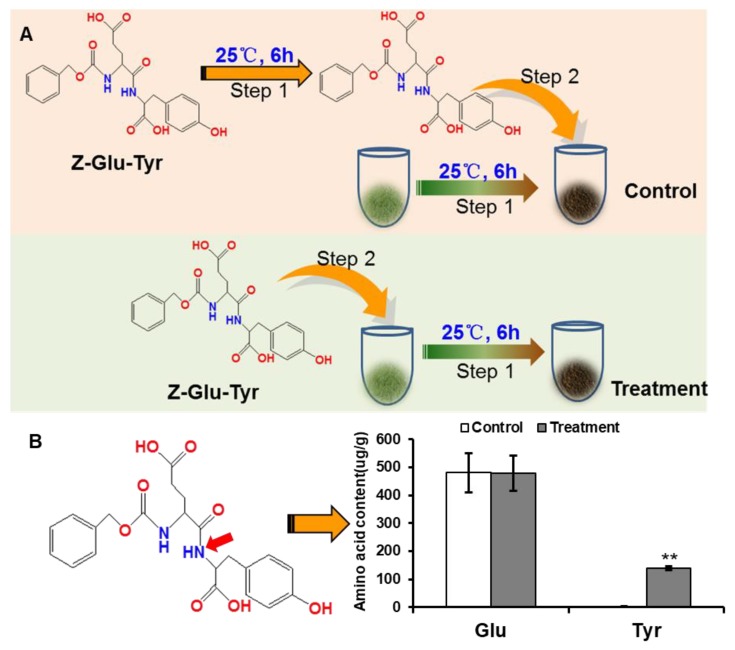
Identification of protein degradation during black tea fermentation with an artificially synthesized dipeptide benzyloxycarbonyl glutamyl-tyrosin (Z-Glu-Tyr). (**A**) Schematic diagram of experimental design. Control: Z-Glu-Tyr and tea leaves powder were fermentation respectively at 25 °C for 6 h, and then mixed to detect the contents of free Glu and Tyr. Treatment: Z-Glu-Tyr and tea leaves powder were mixed first, and then companied fermentation at 25 °C for 6 h step 1, Z-Glu-Tyr and tea leaves powder were fermentation at 25 °C for 6 h respectively (control) or mixed (treatment); step 2, Z-Glu-Tyr mixed with tea leaves powder. (**B**) Changes of Glu and Tyr content after enzymatic reaction during the fermentation. Data shown as the mean ± SD (*n* = 3). ** *p* < 0.01 vs. Control.

**Figure 3:**
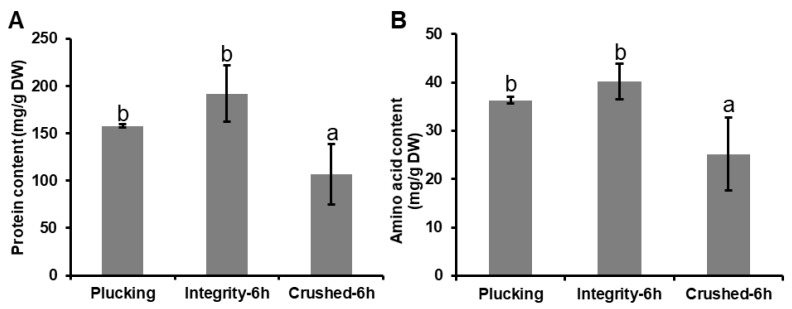
Changes of protein content (**A**) and amino acids content (**B**) in enzymatic reaction of black tea. Bars indicate the means ± S.D. (*n* = 3) of three biological replicates, and bars with different letters are significantly different at *p* ≤ 0.05 according to Duncan’s multiple range test.

Proteins are reportedly broken down into amino acids by enzyme peptidase during the black tea manufacturing process [39,40]. The enzyme reaction is a long process beginning with tea leaf plucking and ending with firing. Particularly during the withering and fermentation processes, black tea leaves make use of sufficient enzyme action [41]. In 1964, Sanderson and Roberts reported that the progressive increase in free amino acids in tea leaves during withering was due to the hydrolytic activity of endogenous peptidase [42]. During the fermentation process, most research has focused on endogenous oxidative enzymes, such as the conversion of selected flavanol combinations to theaflavins and thearubigins [43]. However, less attention has been paid to proteolytic enzymes. Furthermore, previous studies have only detected changes in protein and amino acid contents during the black tea manufacturing process, while direct evidence is lacking. In this study, we selected artificial synthetic dipeptide Z-Glu-Tyr as a representative substrate to directly identify protein degradation into amino acids during black tea fermentation (Figure 2A). Z-Glu-Tyr is a complex with two aromatic moieties, namely, the phenyl group in Z and phenol group in tyrosine, of which the former is more hydrophobic [44]. Therefore, tyrosine is more readily broken down by Z-Glu-Tyr. In this study, Z-Glu-Tyr was added to the crushed tea leaves, and the black tea fermentation step was simulated for 6 h. A larger amount of tyrosine was detected in the tea leaves after fermentation, while tyrosine was not detected in the control. These results suggested that protein degradation occurred during black tea fermentation, and that tyrosine was obtained from the breakdown of Z-Glu-Tyr. Interestingly, the glutamate content showed no significant change in this study (Figure 2B), which might be due to the phenyl group of Z not being able to breakdown glutamate, and the glutamate in group Z cannot be detected. In the simulation of black tea fermentation, compared with the plucked leaves, the protein content was significantly decreased in crushed tea leaves after fermentation for 6 h, without obvious changes in leaf integrity (Figure 3). A crushing process has been designed to simulate the rolling of black tea, with complete disruption of tea leaf cells in this process leading to interactions between substrates and enzymes [45]. The results suggested that tea leaf protein was degraded to free amino acids during the withering, rolling, and fermentation processes. However, the amino acid content did not increase, but decreased (Figure 3). Most free amino acids also showed decreased contents after fermentation for 6 h compared with fresh tea leaves (Table S2). This evidence suggested that the free amino acids were converted into other metabolites during black tea fermentation.

### 3.3. Proteinaceous Amino Acid Metabolism during Black Tea Fermentation

**Figure 4:**
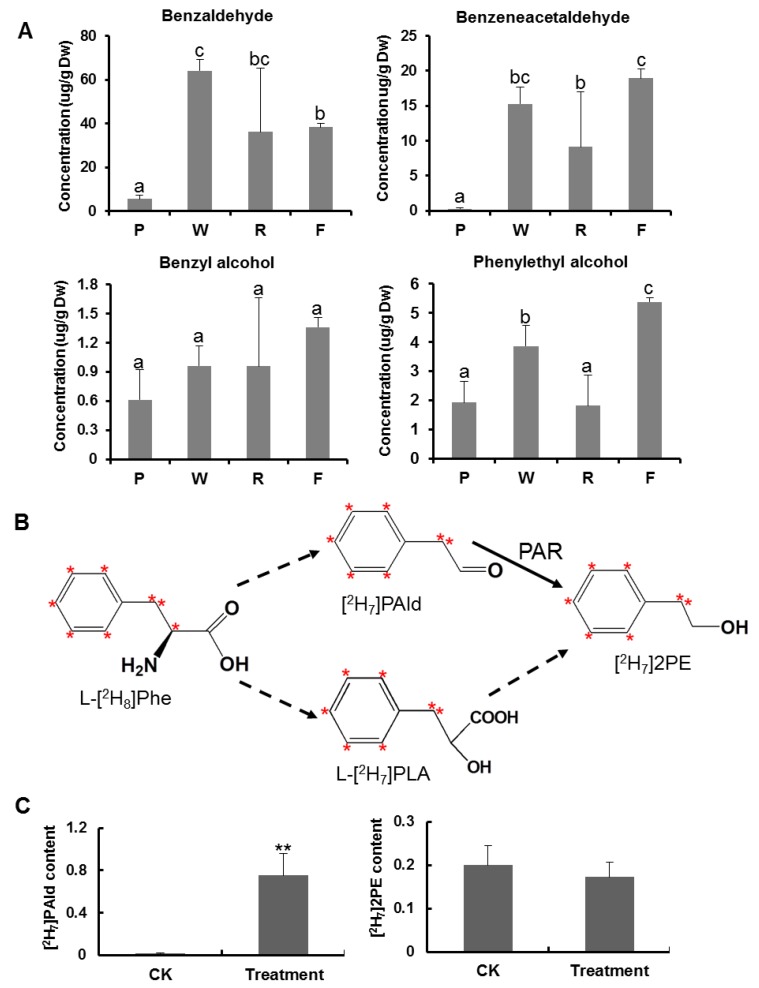
Aroma compound concentrations in phenylalanine degradation pathway Phe, phenylalanine; PAld, phenylacetaldehyde; 2PE, 2-phenylethanol; PAR, phenylacetaldehyde reductase. (**A**) Aroma compound concentrations in phenylalanine degradation pathway during the enzymatic reaction of black tea; (**B**) The known pathway of L-phenylalanine degradation; (**C**) Identification the transformation of L-phenylalanine to aroma compound with [^2^H_8_] L-phenylalanine. Data shown as the mean ± SD (*n* = 3). ** *p* < 0.01 vs. CK. Bars indicate the means ± S.D. (*n* = 3) of three biological replicates, and bars with different letters are significantly different at *p* ≤ 0.05 according to Duncan’s multiple range test.

Free amino acids undergo appreciable changes during the black tea manufacturing process (Table 1). The free amino acid contents decreased overall during fermentation, suggesting that amino acids were converted into other substances. Some amino acids have been reported to increase tea aroma [46]. There are two possible pathways for converting free amino acids into tea aroma compounds or volatile compounds. The first involves combination with orthoquinone, an oxidized form of catechin, which plays an important role in determining the black tea aroma [47,48]. The second pathway, undergone by some amino acids, including phenylalanine, leucine, isoleucine, and valine, involves partial conversion to the aldehydes expected from a Strecker degradation [47]. In this study, we selected phenylalanine as the representative amino acid to investigate free amino acid degradation during black tea fermentation, because the phenylalanine degradation metabolites (including benzaldehyde, benzyl alcohol, and methyl benzoate) contribute to the main aroma properties of black tea flavor [49]. The phenylalanine content decreased significantly during the fermentation stage of black tea manufacture (Table 1). To investigate changes in the volatile compounds during the black tea manufacturing process, samples from five manufacturing stages were analyzed by GC-MS. Four volatile compounds (benzaldehyde, phenylacetaldehyde, benzyl alcohol, and phenylethyl alcohol) involved in the phenylalanine degradation pathway were detected and identified by direct comparison with authentic standards. The concentrations of each compound were calculated based on calibration curves. The results showed that all four volatile compounds had significantly increased contents at the fermentation stage compared with the plucking stage (Figure 4A, Figure S1). Previous reports showed that these four compounds were derived from L-phenylalanine via *trans*-cinnamic acid or directly from L-phenylalanine in tea leaves [49,50]. Kraujalytė et al. (2016) reported that aldehydes were the most abundant group comprising 55% of total identified volatiles in black tea, phenylacetaldehyde, and benzaldehyde respectively contribute honey-like odor and almond odor in black tea [51]. Benzyl alcohol was also the prominent scent compounds in black, and largely contributes to the floral aroma [49].

To further confirm the degradation of phenylalanine to volatile compounds during the fermentation stage, [^2^H_8_] L-phenylalanine was added to the crushed tea leaves and the fermentation step of black tea manufacturing was simulated. After fermentation for 6 h, [^2^H_7_] phenylacetaldehyde ([^2^H_7_] PAld) and [^2^H_7_] 2-phenylethanol ([^2^H_7_] 2PE) were detected (Figure 4C). PAld and 2PE are considered important constituents for black tea quality owing to their rose-like aroma. A previous study showed that 2PE could be derived from L-Phe via the PAld route and PLA route in *Rose* [52]. After fermenting for 6 h, [^2^H_7_] PAld was detected in large quantities, but was not detected in the control. Furthermore, [^2^H_7_] 2PE was detected both in the treatment and control groups, with no significant differences in content (Figure 4C). This suggested that 2PE might be derived from L-Phe via the PLA route (Figure 4B), which can occur rapidly. These results confirmed that free amino acids can be degraded to volatile compounds during black tea fermentation.

## 4. Conclusions

**Figure 5:**
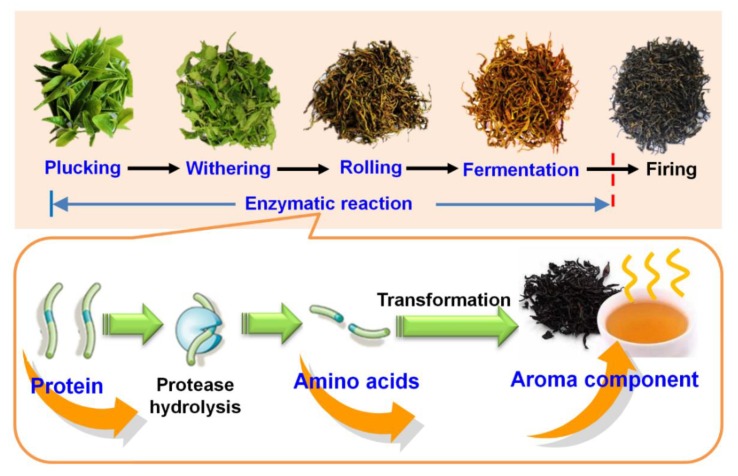
Summary on enzymatic reaction related protein degradation and proteinaceous amino acids metabolism during the black tea (*Camellia sinensis*) manufacturing process.

Our results showed that the decrease in protein concentration during the fermentation stage of black tea manufacture was due to protein degradation (Figure 5). We also confirmed that the proteins were broken down to free amino acids using artificially synthesized dipeptide Z-Glu-Tyr. However, results from the automatic amino acid analyzer showed that, during the black tea fermentation stage, the free amino acid concentrations did not increase, but decreased overall. Metabolites of the amino acid metabolic pathway were detected to have significantly increased contents during fermentation. We also verified that free amino acids were degraded into volatile compounds using a tracer experiment with an isotope precursor. Our results confirmed protein degradation and proteinaceous amino acid metabolism during the fermentation stage of black tea manufacture and provided information that will help black tea manufacturers improve black tea quality.

## Figures and Tables

**Table 1:** Contents of free amino acids in black tea leaves during the manufacturing process.

Amino Acid μg/g (Dry Weight)	Plucking	Withering	Rolling	Fermentation	Drying
P-Ser	146.89 ± 9.21 ^a^	136.29 ± 14.76 ^a^	154.64 ± 2.69 ^a^	156.27 ± 11.46 ^a^	96.39 ± 34.04 ^b^
PEA	79.13 ± 16.46 ^b^	127.90 ± 7.40 ^a^	94.57 ± 6.85 ^b^	50.69 ± 5.75 ^c^	39.06 ± 15.45 ^c^
Asp	13.97 ± 8.92 ^b^	20.61 ± 4.59 ^b^	21.85 ± 4.12 ^ab^	30.57 ± 5.52 ^b^	40.04 ± 14.91 ^a^
Thr	140.04 ± 25.22 ^c^	277.19 ± 6.76 ^a^	264.51 ± 39.46 ^ab^	229.25 ± 15.56 ^b^	130.85 ± 3.96 ^c^
Ser	564.34 ± 53.16 ^c^	880.84 ± 74.88 ^a^	736.48 ± 56.08 ^b^	663.63 ± 46.75 ^b^	383.09 ± 21.37 ^c^
Asn	0.00 ± b	382.22 ± 662.02 ^ab^	678.97 ± 145.24 ^a^	556.45 ± 78.34 ^ab^	391.01 ± 84.43 ^ab^
Glu	2331.12 ± 310.66 ^a^	1609.88 ± 101.01 ^b^	1423.61 ± 22.43 ^bc^	1224.51 ± 96.73 ^c^	773.41 ± 12.54 ^d^
Thea	6874.15 ± 1226.16 ^a^	5284.69 ± 572.10 ^b^	5100.84 ± 110.42 ^b^	4618.89 ± 420.34 ^b^	2559.11 ± 18.16 ^c^
α-AAA	21.32 ± 5.85 ^d^	126.19 ± 20.76 ^a^	114.37 ± 11.77 ^ab^	97.48 ± 10.95 ^b^	52.69 ± 9.79 ^c^
Gly	67.38 ± 11.69 ^a^	39.63 ± 2.61 ^b^	35.06 ± 4.82 ^bc^	17.42 ± 14.47 ^cd^	8.21 ± 7.17 ^d^
Ala	248.51 ± 39.53 ^c^	402.25 ± 12.70 ^b^	480.03 ± 15.91 ^a^	470.10 ± 23.93 ^a^	289.01 ± 29.66 ^c^
α-ABA	15.24 ± 2.67 ^a^	14.70 ± 2.44 ^a^	12.89 ± 1.51 ^ab^	9.07 ± 3.52 ^b^	7.77 ± 3.27 ^b^
Cit	2.88 ± 0.28 ^b^	17.34 ± 7.74 ^a^	13.81 ± 3.77 ^ab^	16.62 ± 7.14 ^a^	10.53 ± 3.15 ^ab^
Val	102.20 ± 21.55 ^d^	581.33 ± 68.70 ^a^	479.27 ± 33.04 ^b^	465.69 ± 22.36 ^b^	282.32 ± 25.48 ^c^
Cys	16.11 ± 10.17 ^c^	107.07 ± 19.84 ^a^	64.65 ± 8.54 ^b^	56.20 ± 16.62 ^b^	21.31 ± 2.85 ^c^
Met	1.92 ± 2.61 ^b^	46.54 ± 39.84 ^a^	10.31 ± 2.71 ^b^	6.55 ± 0.21 ^b^	1.82 ± 0.32 ^b^
Ile	14.38 ± 2.06 ^d^	385.67 ± 41.44 ^a^	327.93 ± 36.95 ^b^	307.94 ± 4.83 ^b^	202.77 ± 14.63 ^c^
Leu	11.58 ± 7.69 ^d^	391.21 ± 24.24 ^a^	338.60 ± 27.36 ^b^	307.84 ± 13.90 ^b^	198.18 ± 15.12 ^c^
Tyr	0.00 ± c	555.68 ± 95.14 ^a^	542.77 ± 21.18 ^a^	498.81 ± 35.46 ^a^	334.49 ± 22.82 ^b^
Phe	45.78 ± 6.33 ^e^	1806.72 ± 162.61 ^a^	1396.06 ± 63.67 ^b^	1220.48 ± 60.33 ^c^	734.05 ± 60.09 ^d^
β-Ala	6.61 ± 0.94 ^b^	39.11 ± 27.67 ^ab^	76.65 ± 38.10 ^a^	56.08 ± 10.26 ^a^	31.48 ± 3.90 ^ab^
β-ABA	6.48 ± 0.84 ^b^	4.37 ± 3.80 ^b^	5.86 ± 5.27 ^b^	23.65 ± 12.82 ^a^	8.90 ± 4.76 ^b^
GABA	2.01 ± 0.53 ^d^	59.97 ± 23.77 ^bc^	91.26 ± 6.45 ^a^	78.41 ± 12.86 ^ab^	42.44 ± 4.54 ^c^
His	9.40 ± 1.27 ^c^	80.51 ± 8.41 ^a^	34.53 ± 4.80 ^b^	15.17 ± 1.19 ^c^	19.49 ± 15.88 ^bc^
3Mehis	-	-	-	10.48 ± 11.16 ^a^	1.43 ± 1.37 ^ab^
1Mehis	2.63 ± 0.29 ^a^	1.97 ± 0.51 ^a^	0.90 ± 0.08 ^a^	3.83 ± 3.88 ^a^	1.17 ± 1.35 ^a^
Trp	37.24 ± 20.68 ^c^	296.47 ± 44.63 ^a^	287.80 ± 102.25 ^a^	174.23 ± 65.19 ^b^	72.30 ± 9.56 ^c^
ORN	91.31 ± 99.49 ^c^	360.88 ± 66.09 ^a^	318.92 ± 42.89 ^ab^	173.48 ± 151.58 ^bc^	13.30 ± 6.04 ^c^
Lys	38.72 ± 17.26 ^d^	368.64 ± 59.13 ^a^	267.90 ± 23.81 ^b^	154.20 ± 53.25 ^c^	56.45 ± 7.42 ^d^
Arg	17.76 ± 5.03 ^a^	42.87 ± 23.45 ^a^	26.61 ± 26.08 ^a^	18.45 ± 8.78 ^a^	15.89 ± 8014 ^a^

Data are expressed as mean ± S.D. (*n* = 3). P-Ser, Phosphoserine; PEA, Phosphorylethanolamine; Asp, Aspartic acid; Thr, Threonine; Ser, Serine; Asn, Asparagine; Glu, Glutamate; Thea, Theanine; α-AAA, α-amino acetic acid; Gly, Glycine; Ala, Alanine; α-ABA, α-Aminobutyric acid; Cit, Citrulline; Val, Valine; Cys, Cystine; Met, Methionine; Ile, Isoleucine; Leu, Leucine; Tyr, Tyrosine; Phe, Phenylalanine; β-Ala, β-Alanine; β-ABA, β-Aminobutyric acid; GABA, γ-Aminobutyric acid; His, Histidine; 3Mehis, 1-Methyl histidine; 1Mehis, 1-Methyl histidine; Trp, Tryptophan; ORN, Ornithine; Lys, lysine; Arg, Arginine. Different means with different letters in the same row are significantly different from each other (*p* ≤ 0.05).

## Supplementary Materials

The following are available online at https://www.mdpi.com/2304-8158/9/1/66/s1. Table S1. The changes of water content in tea leaves during black tea manufacturing process. Table S2: Contents of free amino acids in tea leaves of simulated the black tea fermentation. Figure S1. GC-MS chromatograms of aroma compound in tea leaves during black tea manufacturing process.

## Abbreviations

AA	Amino acids
ANOVA	Analysis of variance
BSA	Bovine serum albumin
DW	Dry weight
GC-MS	Gas chromatography-mass spectrometry
1PE	1-Phenylethanol
2PE	2-Phenylethanol
Z-Glu-Tyr	Benzyloxycarbonyl glutamyl-tyrosin
