# Supplementary material for: Enzymatic Reaction-Related Protein Degradation and Proteinaceous Amino Acid Metabolism during the Black Tea (Camellia sinensis) Manufacturing Process
Source: Foods. 2020 Jan 8;9(1):66. doi: 10.3390/foods9010066 (PMC7022896; doi:10.3390/foods9010066)
Supplement: Supplementary file 1 [file foods-09-00066-s001.pdf]

foods

MDPI

Article

# Enzymatic Reaction-related Protein Degradation and Proteinaceous Amino Acid Metabolism during the Black Tea (*Camellia sinensis*) Manufacturing Process

**Yiyong Chen [1,2,†], Lanting Zeng [2,3,†], Yinyin Liao [2], Jianlong Li [1], Bo Zhou [1], Jinchi Tang [1,*], Ziyin Yang [2,3,*]**

[1] Tea Research Institute, Guangdong Academy of Agricultural Sciences & Guangdong Provincial Key Laboratory of Tea Plant Resources Innovation and Utilization, Dafeng Road 6, Tianhe District, Guangzhou 510640, China; chenyiyong@gdaas.cn (Y.C.); skylong.41@163.com (J.L.); zhoubo@gdaas.cn (B.Z.);

[2] Key Laboratory of South China Agricultural Plant Molecular Analysis and Genetic Improvement & Guangdong Provincial Key Laboratory of Applied Botany, South China Botanical Garden, Chinese Academy of Sciences, Xingke Road 723, Tianhe District, Guangzhou 510650, China; zenglanting@scbg.ac.cn (L.Z.); honey_yyliao@scbg.ac.cn (Y.L.)

[3] Center of Economic Botany, Core Botanical Gardens, Chinese Academy of Sciences, Xingke Road 723, Tianhe District, Guangzhou 510650, China

† These authors contributed equally to this work.

* Correspondence: tangjinchi@126.com; Tel.: +86-20-8516-1049 (J.T.); zyyang@scbg.ac.cn; Tel.: +86-20-3807-2989 (Z.Y.)

Received: 9 October 2019; Accepted: 20 December 2019; Published: 8 January 2020

# Supplementary Information

**Table S1.** The changes of water content in tea leaves during black tea manufacturing process.

| | Plucking | Withering | Rolling | Fermentation | Drying |
|---|---|---|---|---|---|
| Water content (%) | 78.03 ± 0.02 | 56.11 ±0.04 | 51.42 ±0.04 | 50.93 ±0.01 | 5.36 ±0.06 |

**Table S2.** Contents of free amino acids in tea leaves of simulated the black tea fermentation.

| Amino acid | Fresh tea leaves | Fermentation (6h) | |
|---|---|---|---|
| μg/g (Dry weight) | (0h) | Intact | Crushed |
| P-Ser | 130.16 ± 2.96[ab] | 145.18 ± 10.95[a] | 175.85 ± 43.04[a] |
| PEA | 132.48 ± 6.81[b] | 193.80 ± 31.08[a] | 14.47 ± 4.05[c] |
| Asp | 14.67 ± 4.98[b] | 22.83 ± 3.13[a] | 18.73 ± 8.30[ab] |
| Thr | 147.39 ± 43.53[a] | 182.56 ± 25.59[a] | 154.16 ± 38.64[a] |
| Ser | 190.70 ± 15.82[c] | 264.17 ± 13.03[b] | 441.50 ± 52.06[a] |
| Asn | 75.14 ± 19.21[b] | 311.48 ± 33.49[a] | 77.84 ± 49.18[b] |
| Glu | 1220.89 ± 144.98[b] | 1364.41 ± 282.47[b] | 2072.11 ± 423.79[a] |
| Thea | 4546.86 ± 1024.10[a] | 5102.98 ± 681.50[a] | 6097 .05 ± 1681.02[a] |
| $\alpha$-AAA | 29.86 ± 26.61[b] | 82.25 ± 17.51[a] | 11.53 ± 9.91[b] |
| Gly | 35.53 ± 0.81[a] | 34.09 ± 28.49[a] | 40.23 ± 7.11[a] |
| Ala | 101.06 ± 13.44[b] | 125.77 ± 28.12[b] | 274.45 ± 59.18[a] |
| $\alpha$-ABA | 16.12 ± 5.30[a] | 16.89 ± 3.32[a] | 15.43 ± 4.36[a] |
| Cit | 13.30 ± 21.13[a] | 1.83 ± 0.21[a] | 2.98 ± 1.55[a] |
| Val | 119.36 ± 40.01[b] | 407.71 ± 50.03[a] | 70.43 ± 14.30[c] |
| Cys | 30.77 ± 7.73[b] | 149.19 ± 59.09[a] | 11.68 ± 4.36[c] |
| Met | 8.83 ± 6.16[b] | 186.29 ± 124.97[a] | 2.12 ± 0.97[b] |
| Ile | 109.19 ± 38.20[b] | 332.75 ± 119.60[a] | 26.57 ± 10.82[c] |
| Leu | 75.26 ± 15.68[b] | 265.38 ±106.85[a] | 52.39 ± 44.52[b] |
| Tyr | 101.43 ± 10.93[b] | 436.84 ± 85.76[a] | 1.44 ± 2.50[c] |
| Phe | 288.87 ± 21.82[b] | 649.26 ± 82.15[a] | 39.96 ± 9.77[c] |
| β-Ala | 12.81 ± 0.12[b] | 50.83 ± 11.37[a] | 13.4 ± 5.38[b] |
| β-ABA | 4.54 ± 1.27[a] | 20.74 ± 19.62[a] | 20.49 ± 18.33[a] |
| GABA | 22.36 ± 3.61[b] | 120.67 ± 28.39[a] | 4.96 ± 0.82[c] |
| His | 47.98 ± 67.60[a] | 112.74 ± 91.91[a] | 7.65 ± 6.45[a] |
| 1Mehis | 2.60 ± 0.92[a] | 2.25 ± 1.73[a] | 1.56 ± 0.72[a] |
| Trp | 198.88 ± 143.53[a] | 291.67 ± 27.86[ab] | 107.82 ± 122.81[a] |
| ORN | 269.81 ± 221.78[ab] | 511.05 ± 136.14[a] | 92.56 ± 101.36[b] |
| Lys | 149.50 ± 69.70[b] | 323.17 ± 30.37[a] | 50.30 ± 54.02[b] |
| Arg | 75.01 ± 41.28[b] | 154.52 ± 34.90[a] | 22.34 ± 26.52[b] |

Data are expressed as mean ± S.D. (n=3). P-Ser, Phosphoserine. PEA, Phosphorylethanolamine. Asp, Aspartic acid. Thr, Threonine. Ser, Serine. Asn, Asparagine. Glu, Glutamate. Thea, Theanine. $\alpha$-AAA, $\alpha$-amino acetic

acid. Gly, Glycine. Ala, Alanine. $\alpha$-ABA, $\alpha$-Aminobutyric acid. Cit, Citrulline. Val, Valine. Cys, Cystine. Met, Methionine. Ile, Isoleucine. Leu, Leucine. Tyr, Tyrosine. Phe, Phenylalanine. β-Ala, β-Alanine. β-ABA, β-Aminobutyric acid. GABA, $\gamma$-Aminobutyric acid. His, Histidine. 1Mehis, 1-Methyl histidine. Trp, Tryptophan. ORN, Ornithine. Lys, lysine. Arg, Arginine. Different means with different letters in the same row are significantly different from each other (p ≤0.05).

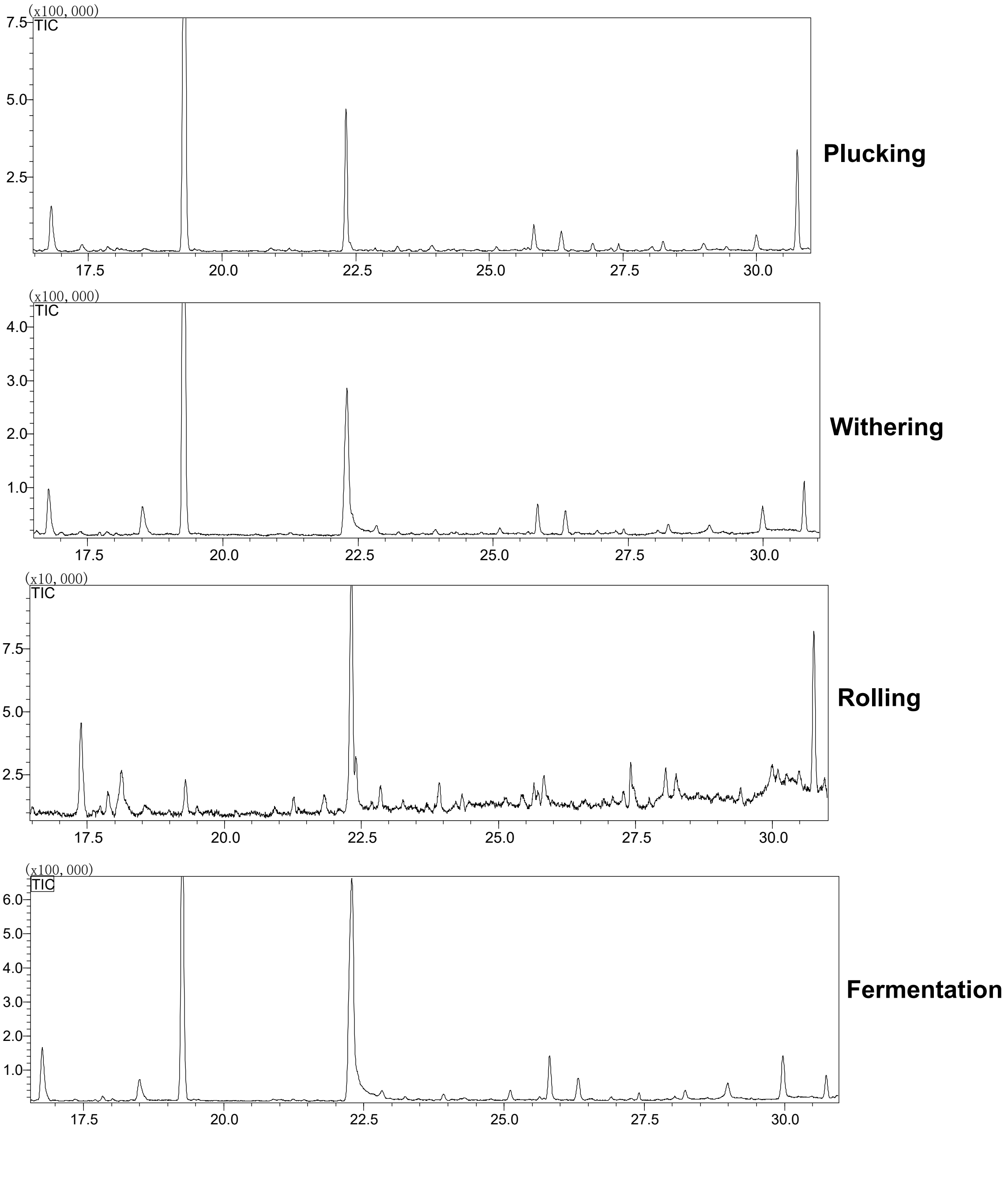

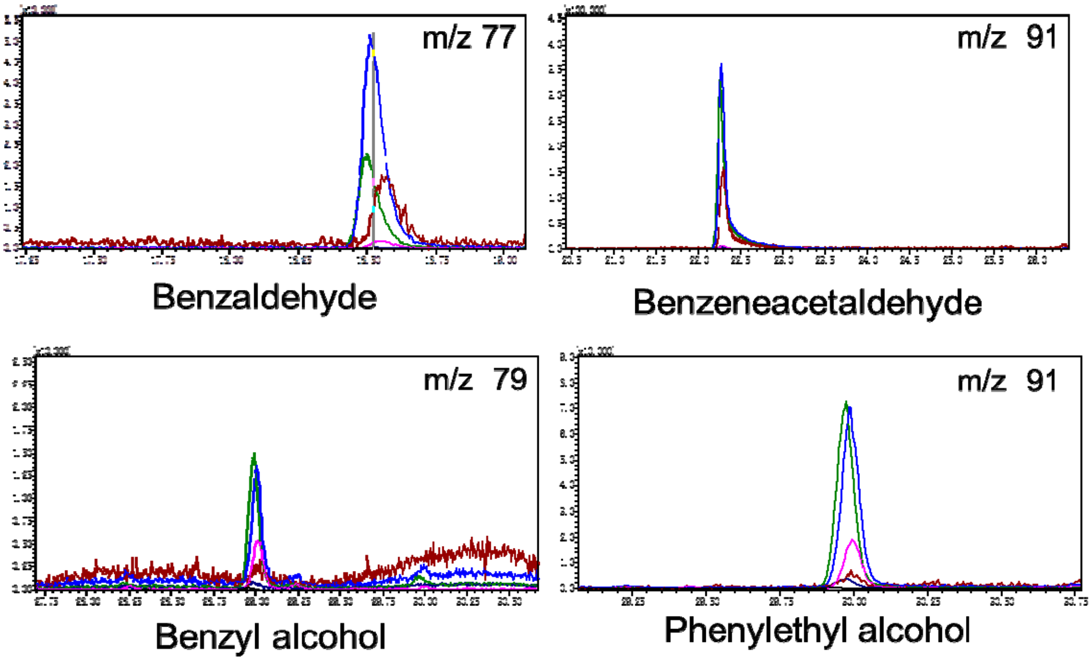


**Figure S1.** GC-MS chromatograms of aroma compound in tea leaves during black tea manufacturing process.
